# The broad autism phenotype predicts child functioning in autism spectrum disorders

**DOI:** 10.1186/1866-1955-5-25

**Published:** 2013-09-22

**Authors:** Christina R Maxwell, Julia Parish-Morris, Olivia Hsin, Jennifer C Bush, Robert T Schultz

**Affiliations:** 1 Center for Autism Research, Children’s Hospital of Philadelphia, 3535 Market Street, Suite 860, Philadelphia, PA 19104, USA; 2 Departments of Pediatrics and Psychiatry, Perelman School of Medicine, University of Pennsylvania, Philadelphia, PA 19104, USA

**Keywords:** Autism spectrum disorders, Broad autism phenotype, Social functioning

## Abstract

**Background:**

Broad autism phenotype (BAP) is a milder expression of the social and communication impairments seen in autism spectrum disorders (ASD). While prior studies characterized the BAP in unaffected family members of probands with ASD, the relationship between parental BAP traits and proband symptomatology remains poorly understood. This study utilizes the Broad Autism Phenotype Questionnaire (BAPQ) in parents and the Social Responsiveness Scale (SRS) in children to examine this connection. We hypothesized that in families affected by ASD, elevated maternal and paternal BAPQ scores would correlate with greater autism symptomatology in diagnosed children. In an extension of prior research, we also explored this relationship in families with typically developing children (TDC).

**Methods:**

Two hundred and forty-five children with ASD, 129 TDC and all parents were recruited as part of a larger study investigating relationships between genes, brain and behavior. The Autism Diagnostic Interview-Revised (ADI-R), Autism Diagnostic Observation Schedule (ADOS) and expert clinical judgment confirmed ASD diagnoses in children. SRS was collected for all children. Parents completed a self-report BAPQ and an informant report BAPQ for their spouse; an average of self-report and informant report for each parent was used in all analyses.

**Results:**

Mothers and fathers of children with ASD had significantly higher rates of BAP traits as compared to parents of TDC. Maternal and paternal BAPQ total scores were not correlated with child IQ in either group. In the ASD group, 10% of mothers and 21% of fathers scored above the established BAP threshold compared to 4% of TDC parents. Crude regression analyses showed that maternal and paternal BAPQ total scores accounted for significant variance in child SRS scores in both ASD (17.1%) and TDC (19.8%) families.

**Conclusions:**

Our results suggest that broad autism symptomatology in parents is moderately associated with their child’s autism symptomatology. This result extended to TDC families, suggesting that the BAPQ and SRS capture subtle, subclinical social variation in both children and adults. These findings could help define multi-generational social impairments in future phenotypic and genetic studies.

## Background

Autism spectrum disorders (ASD) are characterized by impairments in social interaction and communication as well as repetitive behaviors and restricted interests. A milder, subclinical expression of the autism phenotype, known as the broad autism phenotype (BAP), is seen in relatives of children with ASD at a higher rate than in the general population [1]. The BAP encompasses variation in traits including social aloofness, pragmatic language and rigidity [2]. In 2007, Hurley *et al*. published the Broad Autism Phenotype Questionnaire (BAPQ) to capture these subclinical ASD traits [2]. The BAPQ was validated in adults from the general population as well as those that had first-degree relatives with ASD [2,3]. Over the past 5 years, many studies have used the BAPQ to investigate subclinical ASD features, and associate those traits with social and cognitive impairments [3-7].

Early studies using the BAP outlined the social cognitive characteristics of individuals meeting BAP criteria (using neuropsychological interviews) [4,5]. Most recently, a study by Sasson *et al*. revealed that individuals with BAP traits in the social domain measured by the BAPQ display certain social cognitive deficits (for example, facial identity recognition, facial expression recognition and theory of mind) as well as general impairment in social skills as rated during actual social interactions [6]. Gender differences in the number and distribution of BAP traits (males > females) have also been reported using interview and questionnaire assessments of BAP [7,8]. Specifically, in ASD families, the aloof phenotype occurs more frequently in fathers, whereas mothers demonstrate more rigidity.

Although BAP and ASD characteristics are believed to exist on a shared continuum, the relationship between mothers’ and fathers’ trait severity and their children’s social skills has yet to be systematically investigated. The most relevant studies to date examined correlations between children’s scores on the Social Communication Questionnaire (SCQ) and their parent’s BAPQ scores, and found differing results [8,9]. Seidman *et al*. found no relationship between SCQ and parental BAPQ scores, whereas Sasson *et al*. demonstrated that probands had increased SCQ scores when their parents met criteria for BAP [8,9]. Similarly, Klusek *et al*. explored the association between paternal tactfulness and child social and communication impairments using the Autism Diagnostic Interview-Revised (ADI-R) [7]. However, the ADI-R and the SCQ were not developed as continuous measures for indexing symptom severity, and thus may not be the best metrics to correlate with parental characteristics. In contrast, the Social Responsiveness Scale (SRS) is a dimensional measure specifically designed to quantify the social impairments of ASD, and spans many domains including social awareness, social motivation, social communication, mannerisms and social cognition [10]. The SRS has important advantages over measures such as the ADI-R, including a larger item bank, consistent scaling of item response scoring, and much greater item density at mild levels of symptomatology that allow researchers to reliably capture individual differences in the subclinical range.

Prior research has found that children with ASD score significantly higher on the SRS compared to typically developing children (TDC) [10]. In a family study using the child and adult versions of the SRS on participants screened for ASD, Constantino *et al*. found that when both biological parents manifest social impairments, their child has higher levels of social difficulty as well [11]. It is still unknown how the presence and severity of the BAP in parents of children with autism are associated with child social ability, and whether mother and father BAP status are differentially associated with child social ability.

The aims of the present study are twofold. First, we examine the continuous relationship between parental BAPQ scores and SRS scores in offspring (both with and without ASD). Second, we explore the differential contributions of maternal and paternal BAPQ scores to child outcome. The SRS and BAPQ, which allow for a low floor and high ceiling, enable us to effectively examine these relationships in a mixed sample including both ASD and TDC participants. We hypothesized that parental BAPQ scores would positively correlate with child SRS scores in ASD and TDC groups, and that maternal and paternal scores would account for a comparable percentage of variance. This study improves on prior research by collecting BAPQ scores from mothers and fathers as well as SRS ratings on children, allowing us to define the differential contributions of mother and father BAP status to child social functioning.

## Methods

### Sample

Participants included 129 TDC (93 males) and 245 children with ASD (210 males) aged 6 to 18 years old, and their parents (Table 1). These families were part of a larger study investigating relationships between genes, brain and behavior. Families were recruited from the community, in part from *autism*Match (https://autismmatch.org), an online recruitment tool that allows families to receive information about research studies that they may be eligible to participate in. The present subsample included all of those families that had participated in studies at the Center for Autism Research, Children’s Hospital of Philadelphia (Philadelphia, PA, USA), that had complete SRS data for children, and complete BAPQ data for mothers and fathers (both self-report and informant report) at the time of data analyses. Thirty-three families were not included in the study due to incomplete BAPQ. In most cases the biological father was unavailable to complete the questionnaire on himself and the informant report on the biological mother. This study was approved by the Children’s Hospital of Philadelphia Institutional Review Board.

**Table 1:** Child information

**Information**	**ASD**	**TDC**	**Group differences**
Age	10.7 (0.21)	11.4 (0.31)	*t*(372) = −1.9, *P* = 0.050
IQ	98.9 (1.4)	114.2 (1.3)	*t*(350) = −8.1, *P* <0.0001
SRS	80.8 (0.81)	41.1 (0.50)	*t*(363) = 41.4, *P* <0.0001

Mean (standard error) grouped by child information. IQ was obtained using the DAS-II. *ASD* autism spectrum disorders, *DAS-II* Differential Ability Scales, second edition, *SRS*, Social Responsiveness Scale, *TDC* typically developing children.

### Procedures

Trained and supervised research assistants obtained informed consent from all parents, and from children capable of providing assent. The ADI-R [12] and Autism Diagnostic Observation Schedule (ADOS) [13] were completed with one of the research reliable doctoral-level psychologists working at the Center for Autism Research during an interview with a parent or with the child with ASD, respectively. The BAPQ was completed about themselves and their child’s other biological parent, and SRS about their child. Parents of TDC completed the BAPQ about themselves and the child’s other biological parent, and SRS about their child.

All children were administered the Differential Ability Scales, second edition (DAS-II) [14] by supervised masters-level and doctoral-level clinicians as a measure of IQ. Clinicians used the ADOS and ADI-R along with clinical judgment to confirm diagnoses of ASD following Collaborative Programs of Excellence in Autism (CPEA) guidelines. Children with known genetic conditions associated with ASD were excluded from the study. To capture a representative sample of children who have autism, children with ASD and comorbid conditions (for example, anxiety disorders) were included in the study.

The Child and Adolescent Symptom Inventory (CASI) was used to screen for comorbid conditions. Sixty-six percent of participants with ASD had elevated scores on the CASI (*T-*score greater than 65) for attention deficit hyperactivity disorder (ADHD), 22% for oppositional defiant disorder (ODD) and 52% for generalized anxiety disorder (GAD). These percentages are estimates as the CASI is a parental report survey and comorbid conditions were not formally evaluated in the clinic. Inclusion criteria for the TDC group included scores below 11 on the SCQ [15] and absence of a community diagnosis of a DSM-IV Axis I disorder. Families of TDC were excluded from the study if they reported having a first- or second-degree relative with an ASD diagnosis.

### Primary measures of interest

#### BAPQ

Parents completed the BAPQ, a freely available, 36-item measure reporting on social aloofness, rigidity, and pragmatic language in themselves (self-report) and the child’s other biological parent (informant report) [2]. Questions are rated on a 6-point scale from ‘very rarely’ to ‘very often’. Best estimate scores were calculated by averaging parental self-report and informant report scores. Cutoffs for total BAP (male cutoff 3.47; female cutoff 3.19) and BAP subdomains (aloofness: male 4.03; female 3.39; rigidity: male 3.9; female 3.85; and pragmatic language: male 3.09; female 2.9) were defined using the results of a large community-based study that established normative cutoffs at 1.5 standard deviations above the mean in parents of children with ASD and controls [3].

#### SRS

Mothers completed the SRS, a 65-item survey regarding a child’s current and past social behaviors [10] that correlates with the ADI-R [16]. Questions are rated on a scale of 1 to 4 ranging from ‘not true’ to ‘almost always true’. Total raw scores were converted to gender-based *T*-scores, with scores of 59 or less representing the normal range, and scores of 60 and above representing mild to severe social impairment.

### Statistical analyses

Diagnostic group differences in children’s age, IQ and SRS, and differences in parental BAPQ scores were measured using independent samples *t*-tests. Chi-squared and Fisher’s exact tests compared the frequency of parents meeting BAP criteria in ASD versus TDC families. Pearson correlations measured the overall relationship between parental BAPQ scores and child characteristics as indexed by the SRS (Table 2). Linear regression analyses examined the association between child symptomatology (SRS) and total parental BAPQ scores within the ASD and TDC groups separately. Paternal and maternal best estimate BAPQ scores were entered stepwise with child SRS score as the dependent variable.

**Table 2:** Pearson correlations between child characteristics and parental BAPQ collapsed across diagnostic group

**Characteristic**	**Paternal BAPQ**	**Maternal BAPQ**
Age	−0.019	0.032
IQ	0.036	−0.054
SRS	0.367^a^	0.263^a^
ADOS severity	0.034	−0.005

Pearson values displayed. ^a^*P* <0.001. *ADOS* Autism Diagnostic Observation Schedule, *BAPQ* Broad Autism Phenotype Questionnaire, *SRS* Social Responsiveness Scale.

## Results

Diagnostic group differences were found in IQ, SRS and gender ratio, which is consistent with the literature. Groups also differed on chronological age (Table 1). Across diagnostic groups, BAPQ scores in both the mother and father significantly correlated with child SRS score (Table 2). Parental ages at the time in which the child was born did not differ by group (mother: ASD 31.4 years, TDC 31.1 years, *P* = 0.5; father: ASD 33.2 years, TDC 33.4 years, *P* = 0.7).

We investigated the frequency with which parents met BAP criteria according to best estimate scores. Consistent with our hypothesis, more parents scored above BAPQ cutoffs in the ASD group than the TDC group. Specifically, 21% of fathers and 10% of mothers met criteria in the ASD group, whereas 7% of fathers and 1% of mothers met criteria in the TDC group (ASD versus TDC for both maternal and paternal comparisons, *P* <0.001). In order to evaluate the frequency with which parents met criteria for the aloof, rigid and pragmatic language phenotypes, we used thresholds from Sasson *et al*. [3]. In the ASD group, a larger number of fathers met criteria for pragmatic language and rigidity difficulties than mothers. No gender differences were found in TDC families (Table 3). The combined frequency of parents meeting criteria for BAPQ was 26% in ASD families and 8% in TDC families (chi-squared = 22, *P* <0.001). Mean informant and self-report BAPQ scores are presented in Table 4.

**Table 3:** Frequency (in percentage) of parents that meet criteria for the BAPQ by diagnostic group

**Group**	**Subdomain**	**Maternal**	**Paternal**	***P***
ASD (n = 245)	BAP total	9.8%	21.2%	0.634^a^
	Aloof	10.2%	17.1%	0.129^a^
	Rigid	10.6%	21.6%	0.068^a^
	Pragmatic	12.2%	20.0%	0.001^a^
TDC (n = 129)	BAP total	<1%	7%	0.999^b^
	Aloof	6.2%	5.4%	0.999^b^
	Rigid	4.7%	12.4%	0.556^b^
	Pragmatic	3.9%	4.7%	0.999^b^

^a^Chi-squared-test; ^b^Fisher’s exact test. *ASD* autism spectrum disorders, *BAP* broad autism phenotype, *BAPQ* Broad Autism Phenotype Questionnaire, *TDC* typically developing children.

**Table 4:** Mean BAPQ total scores for informant and self-reports

**Parent**	**Report**	**ASD**	**TDC**	***P***
Maternal	Informant	2.60 (0.04)	2.50 (0.05)	0.174
	Self	2.49 (0.04)	2.25 (0.05)	<0.01
Paternal	Informant	2.93 (0.05)	2.54 (0.06)	<0.01
	Self	2.88 (0.05)	2.61 (0.05)	<0.01

Mean BAPQ score (standard error) grouped by diagnosis. *ASD* autism spectrum disorders, *BAPQ* Broad Autism Phenotype Questionnaire, *TDC* typically developing children.

Linear regression analyses measured the relationship between parents’ total BAPQ scores and child ASD symptomatology (Table 5). Paternal and maternal BAPQ scores were entered stepwise in separate equations for the ASD and TDC groups, each predicting child SRS score. The final model was significant in the ASD group, with parental BAPQ scores accounting for 17.1% of the variance in child SRS scores (*F*(2,243) = 24.8, *P* <0.0001). Paternal BAPQ scores entered first, and maternal BAPQ scores accounted for an additional 2.5% of variance. Both maternal and paternal BAPQ scores were significant contributors to explained variance in the final model in ASD families (beta = 0.16, *P* = 0.007 and beta = 0.36, *P* <0.001, respectively).

**Table 5:** Linear regressions with parental BAPQ predicting SRS score in ASD and TDC separately

**Group**	**Model**	**Variable**	**Beta**	***t***	***P***	**R**^**2**^	**ΔR**^**2**^
ASD	1	Paternal	0.382	6.42	<0.001	0.146	0.146
	2	Paternal	0.360	6.08	<0.001	0.171	0.025
		Maternal	0.160	2.71	0.007		
TDC	1	Maternal	0.410	5.06	<0.001	0.168	0.168
	2	Maternal	0.339	3.93	<0.001	0.198	0.030
		Paternal	0.187	2.17	0.032		

Beta is standardized. Model 1 is the single variable listed; model 2 is the combination of both variables. *ASD* autism spectrum disorders, *BAPQ* Broad Autism Phenotype Questionnaire, *SRS* Social Responsiveness Scale, *TDC* typically developing children.

**Figure 1:**
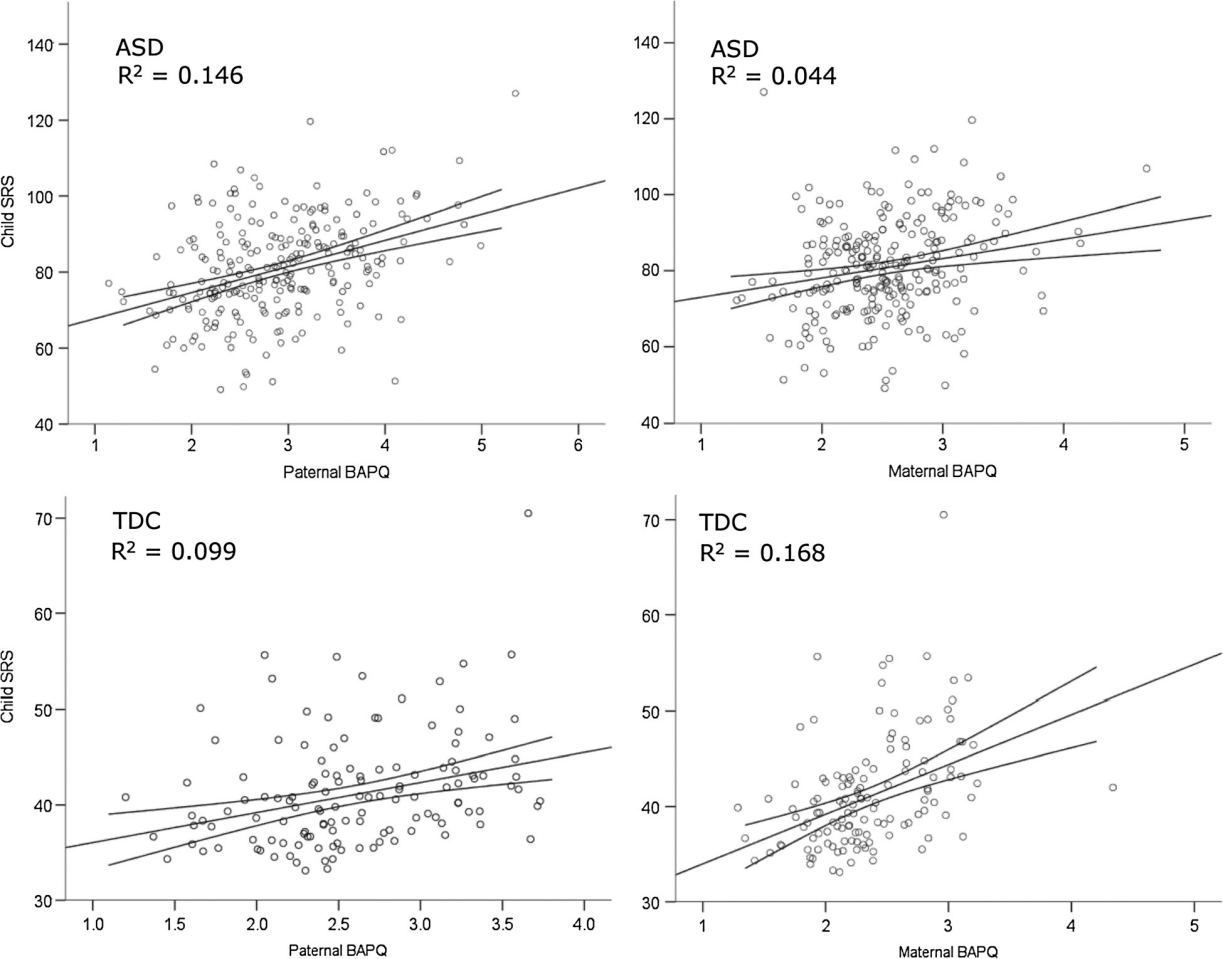
**Scatterplots showing SRS and BAPQ scores by parent (mother or father) and diagnostic group (ASD or TDC).** Note the differences in scale. ASD, autism spectrum disorders; BAPQ, Broad Autism Phenotype Questionnaire; SRS, Social Responsiveness Scale; TDC, typically developing children.

The final model in the TDC group was also significant, with parental BAPQ scores accounting for 19.8% of the variance in child SRS scores (*F*(2,126) = 15.5, *P* <0.0001). In contrast to the ASD group, maternal scores entered into the TDC model first, with paternal BAPQ scores contributing an additional 3% of variance. Both maternal and paternal BAPQ scores accounted for a significant amount of variance in their children’s SRS scores in the final model in TDC families (beta = 0.339, *P* <0.001 and beta = 0.187, *P* = 0.032, respectively; Figure 1).

Our finding that paternal BAPQ accounted for most of the variance in the SRS scores of the ASD group and maternal BAPQ accounted for most of the variance in the SRS scores of the TDC group required further investigation. A Fisher *z*-transformation revealed a significant difference between mother and father BAPQ–SRS correlations in the ASD group (*z* = 2.09, two-tailed *P* = 0.03) but not the TDC group (*z* = −0.97, *P* = 0.4), suggesting a relatively stronger association between paternal BAP traits and child phenotype in ASD families that is not present in TDC families.

In a final analysis, we investigated differences in child SRS when at least one parent met criteria for total BAPQ or exceeded thresholds in at least one subdomain (social aloofness, rigidity and pragmatic language). Independent samples *t*-tests revealed significantly higher child SRS scores in the ASD group when at least one parent met criteria for total BAPQ or exceeded threshold in any subdomain (Table 6). While there was a similar trend in the TDC group, differences reached statistical significance only when parents met criteria for rigid personality or pragmatic language impairment.

**Table 6:** Differences in BAP total and subscores by SRS score

**Group**	**Subdomain**	**SRS BAP absent**	**SRS BAP present**	***P***
ASD (n = 245)	BAP total	77.8 (0.8)	84.3 (1.1)	<0.01
	Aloof	78.2 (0.8)	84.2 (1.2)	<0.01
	Rigid	77.7 (0.8)	83.9 (1.0)	<0.01
	Pragmatic	77.7 (0.8)	84.9 (1.2)	<0.01
TDC (n = 129)	BAP total	40.7 (0.5)	46.1 (2.9)	0.1
	Aloof	40.7 (0.5)	43.7 (1.3)	0.063
	Rigid	40.2 (0.5)	45.8(1.7)	0.004
	Pragmatic	40.6 (0.5)	46.6 (2.7)	0.051

Mean SRS scores (standard error) grouped by parental BAP. BAP absent refers to neither parent meeting criteria for BAP; BAP present refers to at least one parent meeting criteria for BAP. *ASD* autism spectrum disorders, *BAP* broad autism phenotype, *SRS* Social Responsiveness Scale, *TDC* typically developing children.

Since the SRS and BAPQ are reported by the same individuals, it is possible that the associations reported in this study were influenced by a reporter bias. However, it is important to note that the SRS also correlated with the clinician-assessed levels of social impairment as measured by the social domain of the ADOS (spearman’s rho 0.13, *P* = 0.04). There was not, however, a significant correlation between the SRS and the ADOS communication score (spearman’s rho 0.05, *P* = 0.4) or the ADOS repetitive behaviors domain score (spearman’s rho 0.06, *P* = 0.3).

## Discussion

Our primary aim was to examine the continuous relationship between BAPQ scores in parents and social responsiveness in their offspring. In our sample of TDC and ASD participants, there was a strong association between parent and child behavioral phenotypes. As hypothesized, elevated BAPQ scores in the parents of children with ASD was associated with poorer child ASD symptoms as measured by the SRS. This relationship was found in the ASD sample for the composite BAPQ total score, and for each of the aloof, rigid and pragmatic language impairments subdomains of the BAPQ. While this finding is correlational, it might represent a shared genetic liability between parents and offspring. Interestingly, this trend was also present in TDC families, suggesting that these phenotyping measures may capture subtle, subclinical social variation. Future behavioral genetic studies should model the within-family variance to estimate the degree to which genetic and non-genetic factors mediate these relationships.

The second aim of our study was to investigate the differential effects of maternal and paternal BAPQ scores on child social responsiveness. We found that maternal and paternal BAPQ scores were additive independent predictors of social reciprocity in children with ASD. To our knowledge, this is the first study to collect BAPQ scores on both mothers and fathers as well as SRS ratings on children in a large group of families. The idea that father characteristics are more strongly associated with child ASD phenotype than mother characteristics is consistent with the literature [7]. For example, using the modified Personality Assessment Schedule (M-PAS), Klusek *et al*. showed that untactful traits in fathers are associated with increased social and communication impairment in their children with ASD [7].

Our results indicated gender differences in the frequency of parents meeting BAP criteria in the ASD group, with 10% of mothers and 21% of fathers scoring above the BAPQ threshold. These frequencies are generally consistent with other studies showing that 9% to 21% of mothers and 9% to 40% of fathers meet criteria for BAP [2,8,17]. However, our pattern of results contrasts with a recent larger study to determine BAP occurrence in ASD families and the general population [3]. Although Sasson *et al*. reported that a similar percentage of fathers met criteria, they reported a larger percentage of mothers (23%) than the present study. It is not known what differentiated the families participating in each study that might lead to such discrepant frequencies or whether these frequency differences are within the expected range for this maternal characteristic.

While the current study adds to the literature by highlighting associations between parental and child phenotypic expression in a large sample of families with confirmed diagnoses of ASD and typical control children, there are limitations to the current study. The use of questionnaires for both parents and children may result in shared method variance and reporter biases, as each parent completes multiple reports (for example, SRS, BAPQ self-report and BAP spouse reports). For example, a mother with high levels of BAP traits may misrepresent their child’s social functioning on the SRS. To partially address this limitation, we correlated the social subscore on the ADOS with child SRS for the ASD sample, and found that parent report of child social responsiveness was significantly associated with third-party ratings of social impairment. We also showed a lack of correlation between the SRS and ADOS communication and repetitive behaviors subdomains, suggesting that the SRS is a valuable, specific measure of social functioning in children with ASD. However, since typical children were not administered the ADOS, we cannot test whether this association holds across groups. Future research would benefit from third-party ratings of social competence in TDC and all parents. In addition, future studies may include measures such as the adult version of the SRS (SRS-A) for consistency between questionnaires in parent and child (though different measures used here has some virtue, a simple shared method may reduce variance).

A second limitation of the present study is that we do not currently have access to a large variety of descriptive measures of families, such as socioeconomic status, mental and physical health status, parental marital status, and child comorbidities or ethnicity, and thus cannot examine the effect of these variables on BAPQ or SRS scores, or the relationship between them. Therefore, our results were unadjusted and may be biased due to the aforementioned confounds. Despite these limitations, the findings reported here demonstrate significant familial relationships in broad autism spectrum traits, in both cases and controls. These results suggest that the broad autism traits are influenced by common genetic variation and/or non-genetic familial factors, each of which has important implications for understanding the mechanisms that lead to ASD.

## Conclusion

This study adds to the literature by demonstrating a relationship between parental BAP and child social functioning. Our hypothesis that parent BAPQ scores would correlate with child social responsiveness was confirmed in the ASD population but also extended to our TDC cohort, which suggests that these phenotypic measures may be useful in identifying both clinical and subclinical impairments. This approach for characterizing autistic traits as a broad dimension of human behavior has important implications for future behavioral and genetic studies of ASD, and may be applicable to many neurodevelopmental and psychiatric disorders. A dimension of behavior that is continuous and crosses the diagnostic threshold to subsyndromal and normative ranges of functioning might also be effectively used in treatment studies to measure intervention effectiveness, since this requires a finely graded dimensional measure with good range such that it is applicable across the full range of behaviors encountered in a community sample.

## Abbreviations

ADHD: Attention deficit hyperactivity disorder; ADI-R: Autism Diagnostic Interview-Revised; ADOS: Autism Diagnostic Observation Schedule; ASD: autism spectrum disorders; BAP: broad autism phenotype; BAPQ: Broad Autism Phenotype Questionnaire; CASI: Child and Adolescent Symptom Inventory; CPEA: Collaborative Programs of Excellence in Autism; DAS-II: Differential Ability Scales, second edition, GAD, generalized anxiety disorder; IQ: Intelligence quotient; M-PAS: Modified Personality Assessment Schedule; ODD: Oppositional defiant disorder; SCQ: Social Communication Questionnaire; SRS: Social Responsiveness Scale; TDC: Typically developing children.

## Competing interests

All authors have no competing interests to declare, financial or non-financial.

## Authors’ contributions

CRM contributed to the study design, analysis and interpretation of data, and drafting and revising of the manuscript. JPM contributed to the study design, analysis and interpretation of data, and revising of manuscript. OH contributed to the study design, acquisition, analysis and interpretation of data, and revising of manuscript. JCB contributed to the acquisition and interpretation of data, and revising of manuscript. RTS contributed to the study design, analysis and interpretation of data, and revising of manuscript. All authors read and approved the final manuscript.
